# Baby Open Brains: An open-source dataset of infant brain segmentations

**DOI:** 10.1038/s41597-025-05404-y

**Published:** 2025-08-14

**Authors:** Eric Feczko, Sally M. Stoyell, Lucille A. Moore, Dimitrios Alexopoulos, Maria Bagonis, Kenneth Barrett, Brad Bower, Addison Cavender, Taylor A. Chamberlain, Greg Conan, Trevor K. M. Day, Dhruman Goradia, Alice Graham, Lucas Heisler-Roman, Timothy J. Hendrickson, Audrey Houghton, Omid Kardan, Elizabeth A. Kiffmeyer, Erik G. Lee, Jacob T. Lundquist, Carina Lucena, Tabitha Martin, Anurima Mummaneni, Mollie Myricks, Pranav Narnur, Anders J. Perrone, Paul Reiners, Amanda R. Rueter, Hteemoo Saw, Martin Styner, Sooyeon Sung, Barry Tiklasky, Jessica L. Wisnowski, Essa Yacoub, Brett Zimmermann, Christopher D. Smyser, Monica D. Rosenberg, Damien A. Fair, Jed T. Elison

**Affiliations:** 1 Masonic Institute for the Developing Brain, University of Minnesota, Minneapolis, USA; 2 Department of Pediatrics, University of Minnesota, Minneapolis, USA; 3 Institute of Child Development, University of Minnesota, Minneapolis, USA; 4 Washington University, St. Louis, USA; 5 PrimeNeuro, Durham, USA; 6 University of Chicago, Chicago, USA; 7 Oregon Health Science University, Portland, USA; 8 Minnesota Supercomputing Institute, University of Minnesota, Minneapolis, USA; 9 University of Michigan, Ann Arbor, USA; 10 Department of Neuroscience, University of Minnesota, Minneapolis, USA; 11 University of North Carolina, Chapel Hill, USA; 12 Department of Radiology, University of Southern California, Los Angeles, USA; 13 Center for Magnetic Resonance Research, University of Minnesota, Minneapolis, USA

**Keywords:** Neuroscience, Brain, Magnetic resonance imaging

## Abstract

Reproducibility of neuroimaging research on infant brain development remains limited due to highly variable processing approaches. Progress towards reproducible pipelines is limited by a lack of benchmarks such as gold-standard brain segmentations. These segmentations are limited by the difficulty of infant brain segmentations, which require extensive neuroanatomical knowledge and are time-consuming in nature. Addressing this, we constructed the Baby Open Brains (BOBs) Dataset, an open source resource of manually curated and expert reviewed infant brain segmentations. Anatomical MRI data was segmented from 71 infant imaging visits across 51 participants, using both T1w and T2w images per visit. Images showed dramatic differences in myelination and intensities across 1–9 months, emphasizing the need for densely sampled gold-standard segmentations across early life. This dataset provides a benchmark for evaluating and improving pipelines dependent upon segmentations in the youngest populations. As such, this dataset provides a vitally needed foundation for early-life large-scale studies such as HBCD.

## Background & Summary

Processing pipeline variability is a critical factor contributing to reproducibility challenges in neuroimaging research. When the same functional imaging dataset is analyzed by a variety of processing pipelines, different conclusions are drawn depending on which approaches were used^1^. A variety of different processing stream decisions affect final conclusions, including pipeline components on both the structural and functional side^2,3^. To support reproducible neuroimaging research, benchmarks must be identified for best standards and practices. One of these necessary benchmarks is gold standard manually defined brain tissue segmentations^4^.

Nowhere are manually defined segmentations more needed than in studying the first 1000 days of life, a dynamically changing period of brain growth and development^5,6^. 80% of brain growth occurs during the first 1000 days of life, including dramatic synaptogenesis, myelination, and other cellular processes^7–9^. Aggregating over 100,000 participants from over 100 MRI studies, Bethlehem *et al*. found that brain development growth acceleration peaks at 7 months of age, with velocity highest around the first three years of life^5^. Work by Alex *et al*. confirmed this velocity peak and showed that these trajectories of growth are linked to cognitive and motor outcomes at 2 years of age and that these trajectories differ by sociodemographic factors and adverse birth outcomes^6^. This dynamic period of growth complicates accurate cortical and subcortical segmentation^10^. The considerable myelination through the first year of life causes T1-weighted (T1w) scans (which enhance the signal of fatty tissue) and T2-weighted (T2w) scans (which enhance the signal of water) to show a contrast spin-inversion effect during this period^11^. Existing studies remain limited due to protocols that varied considerably in processing mechanisms, including varied early life segmentation atlases^4,12,13^. In this context, an atlas refers to a common set of labels for each brain structure within a whole brain MRI scan; a set of atlases can be used to segment new MRI scans and inform where common structures are across brains. Thus, a researcher can confidently know that they are examining the same brain region in two different children’s brains.

Standardized infant segmentation atlases have become a critical need within research programs. The NIH has already invested $50 + million, and plans to invest hundreds of millions more, in the HEALthy Brain and Child Development (HBCD) study. This study promises to elucidate neurodevelopmental trajectories with unprecedented precision and rigor^14,15^ and overcome sample size limitations highlighted by Marek *et al*.^16^. This fills a critical need for measuring true effect sizes for brain-wide associations relevant to early-life outcomes. Correct structural brain segmentations are essential to this promise, especially during the first 9 months due to the dynamic processes of growth and myelination occurring^17–19^. Thus, an atlas is needed that supports the dynamic changes within this time period. Yet the availability of manually-corrected segmentations from anatomical MRI data across infancy is limited^4^. Such corrections require considerable neuroanatomic expertise, expertise linking MRI landmarks to neuroanatomic borders, and are time-intensive, thus requiring considerable effort.

As field-wide momentum grows for reproducible research standards, a philosophy of open science is a necessary component of research best practices^20^. Without transparent research, factors that contribute to low reproducibility rates cannot be examined. In this context, as underlying manual segmentations are an impactful part of processing pipelines, it stands to reason that these segmentations should themselves be open and transparent. The primary objective of this resource was to construct a set of manually curated and expert reviewed human infant brain segmentations that adhere to FAIR^21^ data principles (Findable, Accessible, Interoperable, and Reusable). This dataset can be used to assess existing pipelines and/or develop new ones, such as the recently presented BIBSNet algorithm that was trained on this dataset^22^. Early life segmentation algorithms already exist within the literature^12,23–33^. However, many lack coverage across the whole-brain (eg. ID-Seg^24^, MANTiS^27^, iSEG challenges^25^, SDM U-net for subcortical^23^, ANUBEX^30^, SegSrgan^29^), use only T1w or only T2w images as inputs (eg. Infant Freesurfer^12^, MCRIB-S^26^, ID-Seg^24^), or are specific to neonatal periods (VINNA^31^) and aren’t reliable across the full first years of life^4^. As well, the underlying training data for those algorithms is often unavailable to the scientific community (iBEAT^32^). Finally, widespread disagreements among researchers can exist even for well-established areas like Wernicke’s area or the hippocampus^34,35^.

Therefore, a lack of high quality, publicly available training data is a major limitation to improved infant segmentation pipelines, which is often pointed out by the developers of these algorithms themselves^24,31^. Making such manual corrections available via open repositories would subject such segmentations to broader exposure and review, improving the rigor and fidelity of the manual segmentations. Indeed, such work has already been performed extensively in adults and even in fetal tissue (ex.^36–40^), and numerous segmentations have been made publicly available via repositories like OpenNeuro^41^.

The Baby Open Brains (BOBs) dataset addresses the need for openly available manually corrected segmentations of MRI data during the earliest periods of life^4^. Such a resource is critical for developers wishing to create processes for accurate automated segmentations. The curation of such a dataset requires considerable neuroanatomic expertise, including knowledge of anatomical MRI landmarks for accurate segmentations. Until now, the labor and considerable effort required to conduct such work has left much of the methods development without a ‘gold standard’ or benchmark dataset. This lack of proper benchmarks has limited the ability for pipeline developers to generalize infant processing pipelines and ensure the effectiveness of different pipelines across infant age groups, which has subsequently led to constrained pipelines tuned for particular ages.

BOBs manual segmentations will provide a benchmark for evaluating and improving automated segmentations. As infant neuroimaging expands, the research community will observe an exponential increase in MRI segmentation approaches. Already at least a half dozen early life segmentation algorithms exist within the literature^12,23–30^; however, few have tested segmentations across the early life age span and that cover the whole-brain and incorporate a wide-breadth of labels beyond gray matter, white matter, and CSF. Combining expert and community review, the BOBs dataset provides a unique foundational benchmark for evaluating and improving image segmentation methods, as well as expanding their scope towards more comprehensive segmentations. Such benchmarks standardize methods development as methods researchers can evaluate segmentation performance and validate tool capability. Such a benchmark standard for performance evaluation facilitates best practices and standards in infant neuroimaging.

These algorithms will form a necessary foundation for early-life large-scale studies such as HBCD. Automated MR processing pipelines specifically designed for early development are necessary to allow large-scale studies such as HBCD to create MR outputs unconfounded by age. With the BOBs resource providing a foundational benchmark to evaluate and improve these processing pipelines, HBCD and other future early-life neuroimaging studies will be well-equipped to provide the promised knowledge of nuanced neurodevelopmental trajectories and their complex environmental interactions.

## Methods

### The dataset is comprised of baby connectome project (BCP) anatomic and segmentation MRI data

The data for the BOBs dataset is pulled from the Baby Connectome Project (BCP), a longitudinal neuroimaging study in infants 0–5 years old. Detailed methodology has been described previously^19^. Briefly, infants were recruited from departmental research participant registries based on both state-wide birth records and the broader communities around the University of North Carolina at Chapel Hill and the University of Minnesota. Infants were eligible for the BCP if they 1) were born at a gestational age of 37–42 weeks, 2) had a birth weight appropriate for gestational age, and 3) had an absence of major pregnancy and delivery complications. Parents provided informed consent and permission for their child’s study participation and data sharing prior to participation. All procedures were approved by the University of North Carolina at Chapel Hill (Study #16-1943) and University of Minnesota Institutional Review Board (SITE00000093). For this dataset, 71 MRI visits with good quality data from infants 1–9 months old scanned at the University of Minnesota were used. Images selected for the dataset represented best quality images based on visual review by the authors, which remains the gold standard for quality assurance in comparison to automated methods^42,43^. Specifically, images were inspected for signs of poor quality such as motion, ghosting, blurriness, ringing, signal drop-off or image cut-offs. MRI data was collected using a 32-channel head coil on a Siemens 3 T Prisma scanner and included high resolution T1w (MPRAGE: TR 2400 ms, TE 2.24 ms, TI 1600 ms, Flip angle 8°, resolution = 0.8 × 0.8 × 0.8 mm3) and T2w (turbo spin-echo sequences: turbo factor 314, Echo train length 1166 ms, TR 3200 ms, TE 564 ms, resolution = 0.8 × 0.8 × 0.8 mm3, with a variable flip angle) structural scans collected during natural sleep.

### Segmentations were initialized using two different segmentation pipelines

As a starting point for manual reviewers, segmentations were run through one of two segmentation pipelines. The first segmentations were initialized from a joint label fusion (JLF) pipeline^44^, and then manually curated. However, such a procedure required many hours of manual curation as these initializations required much coarser edits. Therefore, these initial manual segmentations were used to train “BIBSNet”^22^, a deep neural network built using nnU-Net^45^ and SynthSeg^33^. Using BIBSNet, other segmentations were initialized and then manually curated. Iteratively using BIBSNet prototypes as a starting point saved many hours of work, as the prototypes were much more accurate starting points than the JLF pipeline. In both pipelines, Advanced Normalization Tools (ANTs) was used to perform denoising and N4 bias field correction and T1w and T2w images underwent a rigid-body realignment to remove distortions and improve image quality for the reviewers. Detailed information about preprocessing is referenced in^22^ and on the BIBSNet Github (https://github.com/DCAN-Labs/BIBSnet).

### Markers curated segmentations according to a standard operating protocol

**Fig. 1:**
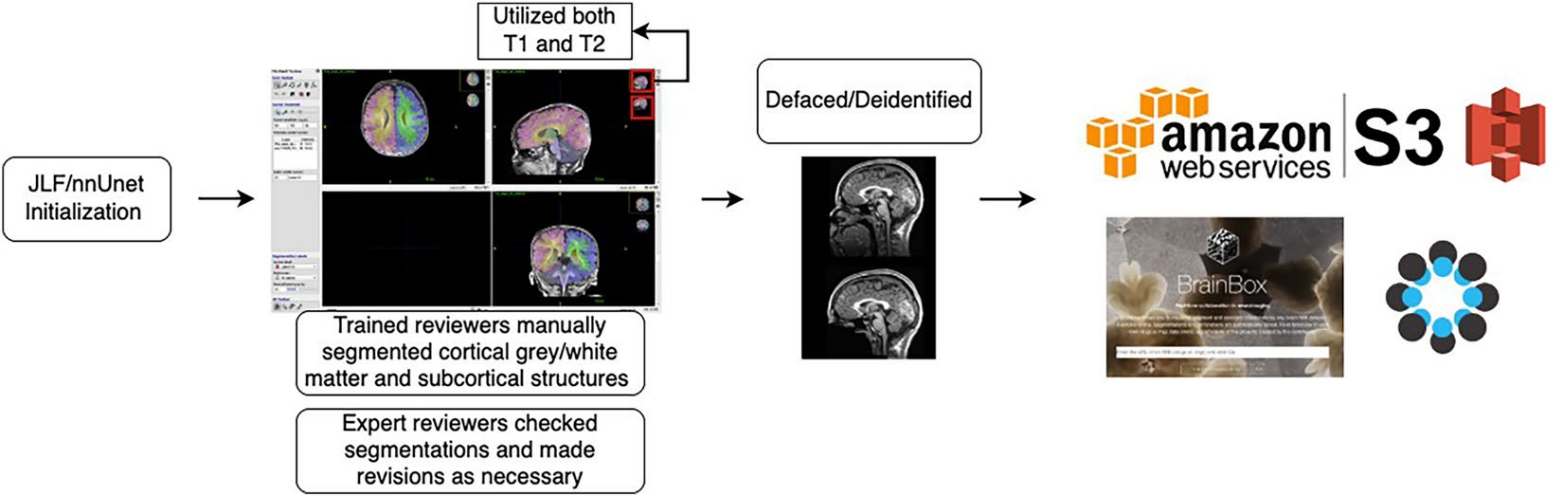
A schematic depicting the process of creating the dataset. Segmentations were initialized with an automated processing pipeline and then manually corrected, utilizing both the T1 and T2 MRI images. Segmentations were then reviewed by expert reviewers who made revisions as necessary. These images were defaced and deidentified, and uploaded to OpenNeuro. OSF acts as a hub to integrate the links to dataset images, protocols, and any other future documentation created as the dataset expands.

A schematic depicting the process of segmentation initialization, correction, and upload is shown in Fig. 1. Markers attended trainings provided by the experts and had regular consultations with expert reviewers throughout the segmentation process. Marker segmentations were reviewed by expert reviewers (EF/SS/JW/DA) and modified as needed. Markers performed image segmentation edits using ITK-SNAP^46^ software. Initialized segmentations were overlaid on top of structural scans and manually edited. Markers utilized both the T1w and T2w scans to determine correct segmentation boundaries, such that there is one segmentation per session. As infant brains in this age range have increasing amounts of myelination in the white matter, referring to both T1w and T2w scans was critical to determining the extent of white matter. For each brain, the cortical surface and the gray-white matter boundary were edited first and reviewed. Subcortical regions were then edited, including the lateral ventricles, inferior lateral ventricles, cerebellum white matter, cerebellum cortex, thalamus, caudate, putamen, pallidum, amygdala, hippocampus, nucleus accumbens, third ventricle, fourth ventricle, and brainstem. Segmentations were done in phases, with the lateral ventricles, third ventricle, and fourth ventricle segmented first, the nucleus accumbens, caudate, putamen, and pallidum second, the brainstem, thalamus, and cerebellum third, and then the amygdala and inferior lateral ventricles last. The hippocampus was segmented separately, either before or after the rest of the subcortical segmentations. Definitions for the boundaries of these regions were pulled from previously published definitions^47–49^. A full SOP of subcortical boundaries was created (See Supplemental Information) and can be found on the OSF site^50^ as well as the ReadTheDocs page (https://bobsrepository.readthedocs.io).

### Approved anatomic MRI data were deidentified and defaced

Final data was stripped of identifying information and formatted into BIDS format. To deface images, T1w and T2w images were run through PyDeface using MNI infant templates as well as a custom infant mask (https://cdnis-brain.readthedocs.io/deidentification/), which masked out facial features from the scans. Final deidentified and defaced images and segmentations were version controlled with DataLad to enable data provenance.

## Data Records

### The BOBs dataset is available on OpenNeuro, with 71 BCP visits spanning 1–9 months of age

**Fig. 2:**
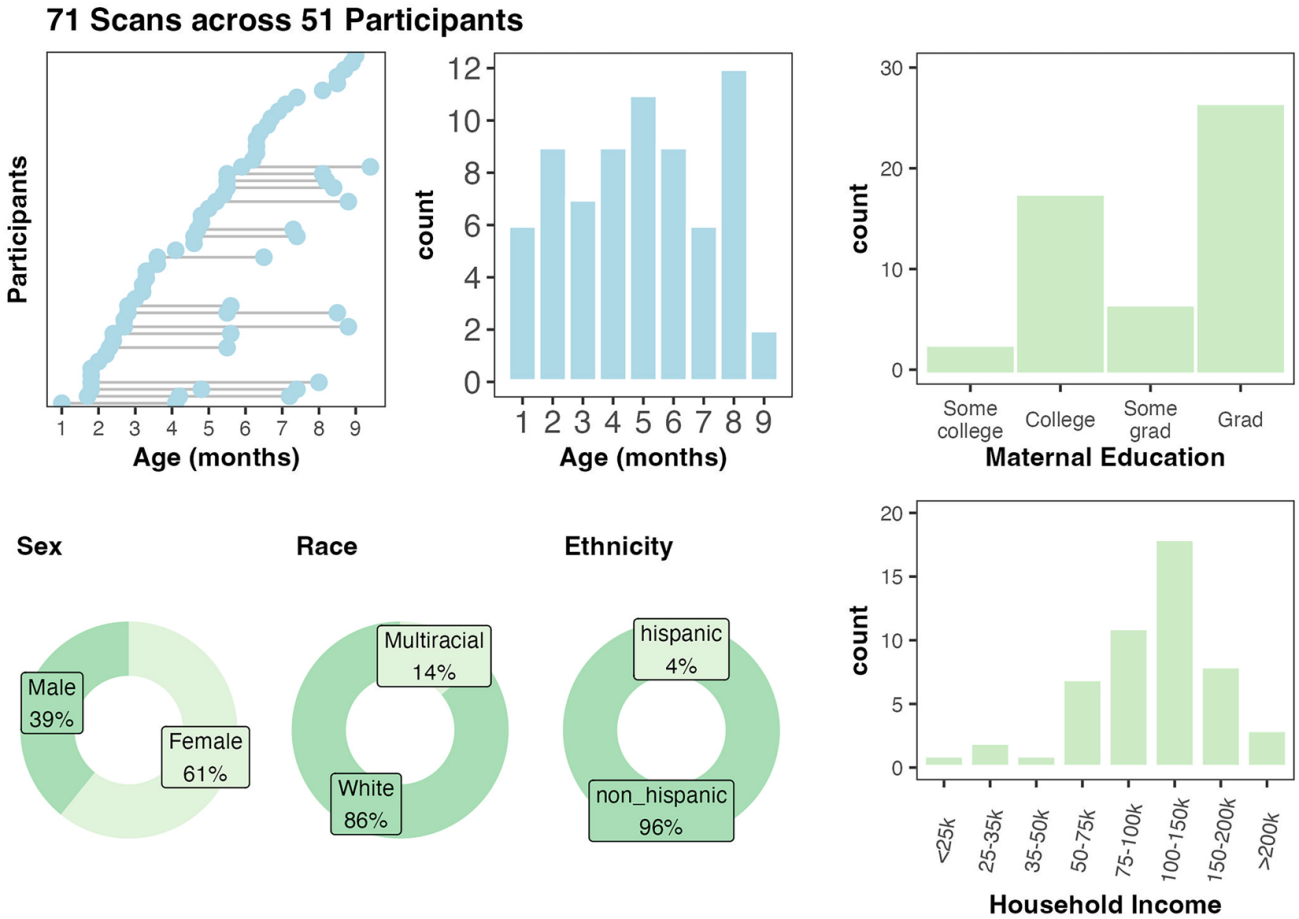
71 scans across 51 participants make up the segmentations in the dataset. All come from the UMN site of the Baby Connectome Project (BCP), and span 1–9 mo. The sample demographics skew white, non-hispanic, and well-resourced.

The BOBs dataset is available on OSF^50^ and OpenNeuro^51^. In total, segmentations were manually curated from 71 imaging visits across 51 participants. Of the 51 participants, 34 participants contributed one scan visit, 14 contributed 2 scans, and 3 contributed 3 scans to this set of segmentations. The age at scan ranged from 1–9 months old, with at least 6 scans at each month 1–8 (Fig. 2). The demographics of the dataset participants skewed White, non-Hispanic, and well-resourced (Fig. 2), with 82% of the sample identifying as White, non-Hispanic and 96% of mothers having at least a college degree. The demographics of the 51 participants pulled for the dataset did not differ statistically from the full BCP neuroimaging sample (N = 901 visits across 383 participants). Select neurodevelopmental measures, including the Mullen Scales of Early Learning, the Vineland Adaptive Behavior Scales, and subscales from the Infant Behavior Questionnaire - Revised, showed no differences between dataset participants and the full BCP sample as well (Table 1), suggesting that participants in this dataset can be considered representative of the larger BCP sample.

**Table 1:** No differences were seen on selected neurodevelopmental scores between the participants selected for the BOBs dataset and the full BCP sample from which they were selected.

	BOBs N = 71 visits (across 51 subjects)	BCP N = 901 visits (across 383 subjects)	p-value
Mullen Early Learning Composite Score (SD)	103.09 (12.67)	104.98 (14.69)	0.34
Vineland Adaptive Behavior Composite (SD)	96.96 (7.66)	99.26 (9.26)	0.07
Infant Behavior Questionnaire - Revised: Smiling and Laughing (SD)	4.61 (0.98)	4.59 (1.10)	0.93
Infant Behavior Questionnaire - Revised: Fear (SD)	2.35 (0.82)	2.19 (0.82)	0.29
Infant Behavior Questionnaire - Revised: Duration of Orienting (SD)	3.64 (1.12)	3.65 (1.11)	0.96

For the Vineland and IBQ-R, as differences are seen in scores across ages, BOBs participants were compared to only those in the BOBs age range (Vineland: N = 127; IBQ-R: N = 77). For the Mullen, the age standardized composite score was used, so BOBs participants were compared to all BCP participants (N = 721 BCP visits with Mullen scores). There were also no differences when just compared between those in the BOBs age range (N = 127).

### The current BOBs dataset is comprised of FreeSurfer-style segmentations for infants

These segmentations comprise cerebral gray/white matter and 23 subcortical structures. Uploaded segmentations went through several review stages before final approval, including at least one expert reviewer manually checking the segmentation. Leveraging both a T1w and T2w, care was taken to label white matter both affected and unaffected by the contrast spin-inversion effect. Diverging from FreeSurfer labels, the ventral thalamic boundary that separates thalamus from ventral diencephalon was defined by the hypothalamic sulcus^52^. The hippocampal label was used to define the hippocampus proper, excluding the formation at the tail along the lingual gyrus, in order to be consistent with other infant literature^53^. While we think evaluating whether the SOP is “right” or “wrong” may be beyond the scope of this paper, we chose such definitions in order to be more consistent with prior infant MRI literature^53^. We welcome the community to inspect and refine existing segmentations to ensure that the “gold standard” benchmarks reflect a community gold standard.

### The BOBs dataset follows BIDS formatting standards

Data within the dataset follows the BIDS formatting standards^54,55^. Each subject folder contains one or more session folders. The “anat” subdirectory within each session folder contains the T1w and T2w image files, the associated segmentation file, and corresponding json files containing metadata for each file. In addition to the subject folders, the directory contains a “dataset_description.json” file, containing a description of the dataset, a “dseg.tsv” file containing a lookup table of segmentation label numbers and names, and a phenotype folder with a “sessions.json” and “sessions.tsv” that contain a list of ID numbers, session, chronological age, gestational age at birth, and sex of the participants in the dataset. The dataset also includes two non-BIDs standard files, “index.html”, a list of links to download individual files, and “V1.0.zip”, a zipped version of the entire repository, that are included for ease of access. File organization can also be found on the BOBs ReadTheDocs page.

## Technical Validation

### Manual segmentations show massive qualitative improvement over initial Joint Label Fusion segmentations

**Fig. 3:**
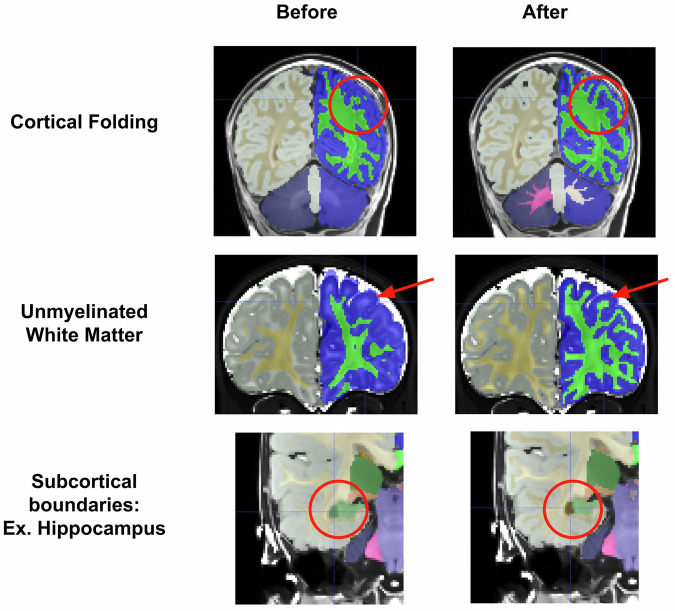
Manual segmentations show massive improvements over initial JLF segmentations. Three cases are demonstrated, showing that reviewers were able to correct errors such as cortical folding patterns, missing unmyelinated white matter, and incorrect subcortical boundaries.

**Fig. 4:**
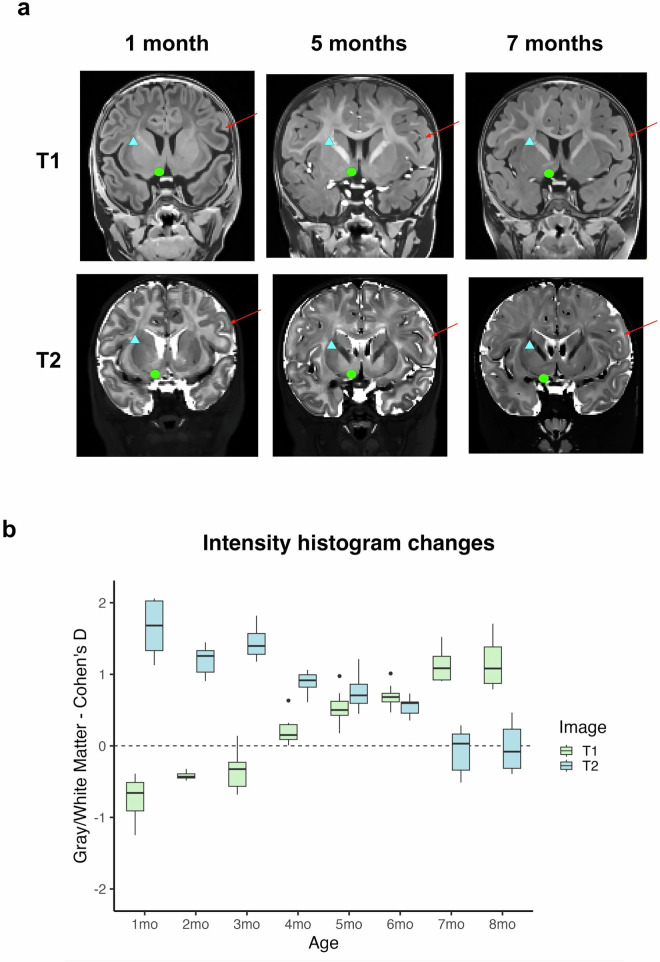
T1w and T2w images show dramatic developmental differences across the age range considered. (**a**) The selected images are from the same participant at three different ages, clearly depicting the transition from unmyelinated to myelinated white matter, and the differing image contrast intensities in the T1w vs. T2w at each age. Red arrows point out cortical gray/white matter changes, blue triangles point out internal capsule white matter changes, and green circles point out nucleus accumbens region changes (**b**) Cohen’s *d* values of white-gray matter differentiation are plotted for T1w and T2w MRI images. Considering both the T1w and the T2w images at this age group is critical to fully capture the white matter and subcortical boundaries.

Compared to initial Joint Label Fusion segmentations, created from the DCAN infant-ABCD-BIDS pipeline^56^, manual segmentations show dramatic qualitative improvements. Initial segmentations had three major types of errors that were corrected by markers (see Fig. 3). First, initial segmentations often created major errors in cortical folding patterns (Fig. 3 top). The initial model may not account for differences between infant and adult image intensity, and this model failure may drive folding pattern segmentation errors. These errors required intensive edits to correct the basic gyri and sulci patterns. Additionally, due to the contrast spin inversion occurring at this age from myelination processes, labeling the full extent of unmyelinated white matter required extensive manual segmentation (Fig. 3 middle). Automated segmentations often miss unmyelinated white matter, especially along the lateral surface of the brain where myelination processes occur later in development. Finally, as exemplified in Fig. 4, subcortical regional intensities change dramatically over this time period, and thus subcortical regional boundaries often needed refining (Fig. 3 bottom).

### Dynamic brain development in infancy requires dense sampling and segmentations utilizing both T1w and T2w images

As infant brains in this age range have increasing amounts of myelination in the white matter^17^, referring to both T1w and T2w scans was critical to determining the extent of white matter. This brain growth is exemplified in a single infant in our dataset across three ages in Fig. 4a. In this infant, there is visually dramatic development of image contrast within and across brain structures. This early time period shows rapid myelination, such that the older ages show much more myelinated white matter, especially along the major white matter tracts. Most dramatically at 5 months in this infant, there is an abundance of unmyelinated white matter that can be easily seen on the T2w image, but would be easily missed on the T1w image. Regardless of the cause, these developmental changes require considering both the T1w and the T2w images at this age group to fully capture the white matter and subcortical boundaries. This was especially critical in subcortical regions such as the basal ganglia, where boundaries might only be visible in either the T1w or the T2w, but not both. The symbols on each of the images exemplify regions that are better served by examining the T1w or the T2w but not both, such as the basal ganglia.

As the largest manually curated human infant brain segmentation dataset for the critical 1–9 month age range, the BOBs dataset proved vital in developing BIBSnet^22^. BIBSnet is an automated segmentation pipeline necessary for HBCD MRI data preprocessing, and critical for infant pipeline development. The BOBs dataset’s critical role in developing BIBSnet establishes further external technical validation for the dataset. Prior efforts towards developing automated segmentation pipelines lacked densely sampled, manually labeled training data that would be critical for early-life longitudinal studies like HBCD^4,14,17–19^. For example, the Developing Human Connectome Project (dHCP) provides extensive anatomical segmentations that are largely restricted to neonatal and preterm infants^28,57^. Such segmentations are derived from the T2w but do not use the T1w; while they can be used to develop automated segmentation pipelines^23,29,30^, such pipelines may fail to generalize beyond the neonatal period. The Infant Freesurfer dataset comprises data from a dozen infant sessions through the first two years of life^58^, and helped develop Infant Freesurfer^12^, but lacks the participant density of the BOBs dataset.

## Usage Notes

In addition to the repository dataset on OpenNeuro, BOBs is available at https://bobsrepository.s3.amazonaws.com/index.html. More information and additional download links are available on our ReadTheDocs page. The dataset was also linked to BrainBox (https://brainbox.pasteur.fr/), which allows users to review the dataset online.

## Supplementary information

Subcortical Segmentation SOPs

## Data Availability

All code used in this manuscript is available publicly as cited in the manuscript or available at https://github.com/sallystoyell/BOBs_manuscript.

## References

[1] Botvinik-Nezer, R. *et al*. Variability in the analysis of a single neuroimaging dataset by many teams. *Nature* **582**, 84–88 (2020). 32483374 10.1038/s41586-020-2314-9 PMC7771346

[2] Glasser, M. F. *et al*. The minimal preprocessing pipelines for the Human Connectome Project. *Neuroimage* **80**, 105–124 (2013). 23668970 10.1016/j.neuroimage.2013.04.127 PMC3720813

[3] Li, X. *et al*. Moving Beyond Processing and Analysis-Related Variation in Neuroscience. *bioRxiv* 2021.12.01.470790 10.1101/2021.12.01.470790 (2024).

[4] Dufford, A. J. *et al*. Un)common space in infant neuroimaging studies: A systematic review of infant templates. *Hum. Brain Mapp.* **43**, 3007–3016 (2022). 35261126 10.1002/hbm.25816 PMC9120551

[5] Bethlehem, R. A. I. *et al*. Brain charts for the human lifespan. *Nature* **604**, 525–533 (2022). 35388223 10.1038/s41586-022-04554-y PMC9021021

[6] Alex, A. M. *et al*. A global multicohort study to map subcortical brain development and cognition in infancy and early childhood. *Nat. Neurosci.* **27**, 176–186 (2024). 37996530 10.1038/s41593-023-01501-6 PMC10774128

[7] Knickmeyer, R. C. *et al*. A structural MRI study of human brain development from birth to 2 years. *J. Neurosci.* **28**, 12176–12182 (2008). 19020011 10.1523/JNEUROSCI.3479-08.2008 PMC2884385

[8] Stiles, J. & Jernigan, T. L. The basics of brain development. *Neuropsychol. Rev.* **20**, 327–348 (2010). 21042938 10.1007/s11065-010-9148-4 PMC2989000

[9] Gao, W. *et al*. Temporal and spatial development of axonal maturation and myelination of white matter in the developing brain. *AJNR Am. J. Neuroradiol.* **30**, 290–296 (2009). 19001533 10.3174/ajnr.A1363 PMC2640448

[10] Mhlanga, S. T. & Viriri, S. Deep learning techniques for isointense infant brain tissue segmentation: a systematic literature review. *Front. Med.* **10**, 1240360 (2023). 10.3389/fmed.2023.1240360 PMC10773803 38193036

[11] Saunders, D. E. *et al*. Magnetic resonance imaging protocols for paediatric neuroradiology. *Pediatric Radiology* **37**, 789–797 (2007). 17487479 10.1007/s00247-007-0462-9 PMC1950216

[12] Zöllei, L., Iglesias, J. E., Ou, Y., Grant, P. E. & Fischl, B. Infant FreeSurfer: An automated segmentation and surface extraction pipeline for T1-weighted neuroimaging data of infants 0–2 years. *Neuroimage* **218**, 116946 (2020). 32442637 10.1016/j.neuroimage.2020.116946 PMC7415702

[13] Gousias, I. S. *et al*. Magnetic resonance imaging of the newborn brain: manual segmentation of labelled atlases in term-born and preterm infants. *Neuroimage* **62**, 1499–1509 (2012). 22713673 10.1016/j.neuroimage.2012.05.083

[14] Volkow, N. D., Gordon, J. A. & Freund, M. P. The Healthy Brain and Child Development Study-Shedding Light on Opioid Exposure, COVID-19, and Health Disparities. *JAMA Psychiatry* **78**, 471–472 (2021). 33295951 10.1001/jamapsychiatry.2020.3803

[15] Dean, D. C. 3rd *et al*. Quantifying brain development in the HEALthy Brain and Child Development (HBCD) Study: The magnetic resonance imaging and spectroscopy protocol. *Dev. Cogn. Neurosci.* **70**, 101452 (2024). 39341120 10.1016/j.dcn.2024.101452 PMC11466640

[16] Marek, S. *et al*. Reproducible brain-wide association studies require thousands of individuals. *Nature* **603**, 654–660 (2022). 35296861 10.1038/s41586-022-04492-9 PMC8991999

[17] Dubois, J., Hertz-Pannier, L., Dehaene-Lambertz, G., Cointepas, Y. & Le Bihan, D. Assessment of the early organization and maturation of infants’ cerebral white matter fiber bundles: a feasibility study using quantitative diffusion tensor imaging and tractography. *Neuroimage* **30**, 1121–1132 (2006). 16413790 10.1016/j.neuroimage.2005.11.022

[18] Shi, F. *et al*. Infant brain atlases from neonates to 1- and 2-year-olds. *PLoS One* **6**, e18746 (2011). 21533194 10.1371/journal.pone.0018746 PMC3077403

[19] Howell, B. R. *et al*. The UNC/UMN Baby Connectome Project (BCP): An overview of the study design and protocol development. *Neuroimage* **185**, 891–905 (2019). 29578031 10.1016/j.neuroimage.2018.03.049 PMC6151176

[20] Nosek, B. A. *et al*. SCIENTIFIC STANDARDS. Promoting an open research culture. *Science* **348**, 1422–1425 (2015). 26113702 10.1126/science.aab2374 PMC4550299

[21] Wilkinson, M. D. *et al*. The FAIR Guiding Principles for scientific data management and stewardship. *Sci Data* **3**, 160018 (2016). 26978244 10.1038/sdata.2016.18 PMC4792175

[22] Hendrickson, T. J. *et al*. BIBSNet: A Deep Learning Baby Image Brain Segmentation Network for MRI Scans. *bioRxiv*10.1101/2023.03.22.533696 (2023). 10.1016/j.dcn.2026.101706 PMC13122235 42013743

[23] Chen, L. *et al*. An attention-based context-informed deep framework for infant brain subcortical segmentation. *Neuroimage* **269**, 119931 (2023). 36746299 10.1016/j.neuroimage.2023.119931 PMC10241225

[24] Wang, Y. *et al*. ID-Seg: an infant deep learning-based segmentation framework to improve limbic structure estimates. *Brain Inform* **9**, 12 (2022). 35633447 10.1186/s40708-022-00161-9 PMC9148335

[25] Sun, Y. *et al*. Multi-Site Infant Brain Segmentation Algorithms: The iSeg-2019 Challenge. *IEEE Trans. Med. Imaging* **40**, 1363–1376 (2021). 33507867 10.1109/TMI.2021.3055428 PMC8246057

[26] Adamson, C. L. *et al*. Parcellation of the neonatal cortex using Surface-based Melbourne Children’s Regional Infant Brain atlases (M-CRIB-S). *Sci. Rep.* **10**, 4359 (2020). 32152381 10.1038/s41598-020-61326-2 PMC7062836

[27] Beare, R. J. *et al*. Neonatal Brain Tissue Classification with Morphological Adaptation and Unified Segmentation. *Front. Neuroinform.* **10**, 12 (2016). 27065840 10.3389/fninf.2016.00012 PMC4809890

[28] Makropoulos, A. *et al*. The developing human connectome project: A minimal processing pipeline for neonatal cortical surface reconstruction. *Neuroimage* **173**, 88–112 (2018). 29409960 10.1101/125526 PMC6783314

[29] Delannoy, Q. *et al*. SegSRGAN: Super-resolution and segmentation using generative adversarial networks - Application to neonatal brain MRI. *Comput. Biol. Med.* **120**, 103755 (2020). 32421654 10.1016/j.compbiomed.2020.103755

[30] Chen, J. V. *et al*. Automated neonatal nnU-Net brain MRI extractor trained on a large multi-institutional dataset. *Sci. Rep.* **14**, 4583 (2024). 38403673 10.1038/s41598-024-54436-8 PMC10894871

[31] Henschel, L., Kügler, D., Zöllei, L. & Reuter, M. VINNA for Neonates–Orientation Independence through Latent Augmentations. *arXiv [cs.CV]* (2023). 10.1162/imag_a_00180 PMC11576933 39575178

[32] Wang, L. *et al*. iBEAT V2.0: a multisite-applicable, deep learning-based pipeline for infant cerebral cortical surface reconstruction. *Nat. Protoc.* **18**, 1488–1509 (2023). 36869216 10.1038/s41596-023-00806-x PMC10241227

[33] Billot, B. *et al*. SynthSeg: Segmentation of brain MRI scans of any contrast and resolution without retraining. *arXiv [eess.IV]* (2021). 10.1016/j.media.2023.102789 PMC10154424 36857946

[34] Tremblay, P. & Dick, A. S. Broca and Wernicke are dead, or moving past the classic model of language neurobiology. *Brain Lang.* **162**, 60–71 (2016). 27584714 10.1016/j.bandl.2016.08.004

[35] Wisse, L. E. M. *et al*. A harmonized segmentation protocol for hippocampal and parahippocampal subregions: Why do we need one and what are the key goals? *Hippocampus* **27**, 3–11 (2017). 27862600 10.1002/hipo.22671 PMC5167633

[36] Caviness, V. S. Jr, Filipek, P. A. & Kennedy, D. N. Magnetic resonance technology in human brain science: blueprint for a program based upon morphometry. *Brain Dev.* **11**, 1–13 (1989). 2646959 10.1016/s0387-7604(89)80002-6

[37] Kennedy, D. N., Filipek, P. A. & Caviness, V. R. Anatomic segmentation and volumetric calculations in nuclear magnetic resonance imaging. *IEEE Trans. Med. Imaging* **8**, 1–7 (1989). 18230494 10.1109/42.20356

[38] Goldstein, J. M. *et al*. Cortical abnormalities in schizophrenia identified by structural magnetic resonance imaging. *Arch. Gen. Psychiatry* **56**, 537–547 (1999). 10359468 10.1001/archpsyc.56.6.537

[39] Seidman, L. J. *et al*. Thalamic and amygdala-hippocampal volume reductions in first-degree relatives of patients with schizophrenia: an MRI-based morphometric analysis. *Biol. Psychiatry* **46**, 941–954 (1999). 10509177 10.1016/s0006-3223(99)00075-x

[40] Payette, K. *et al*. An automatic multi-tissue human fetal brain segmentation benchmark using the Fetal Tissue Annotation Dataset. *Sci Data* **8**, 167 (2021). 34230489 10.1038/s41597-021-00946-3 PMC8260784

[41] Gorgolewski, K., Esteban, O., Schaefer, G., Wandell, B. & Poldrack, R. OpenNeuro—a free online platform for sharing and analysis of neuroimaging data. *Organization for human brain mapping. Vancouver, Canada* **1677** (2017).

[42] Taylor, P. A., Etzel, J., Glen, D. R. & Reynolds, R. C. *Demonstrating Quality Control (QC) Procedures in fMRI*. (Frontiers Media SA, 2023). 10.3389/fnins.2023.1205928 PMC10264898 37325035

[43] White, T. *et al*. Automated quality assessment of structural magnetic resonance images in children: Comparison with visual inspection and surface-based reconstruction. *Hum. Brain Mapp.* **39**, 1218–1231 (2018). 29206318 10.1002/hbm.23911 PMC6866370

[44] Wang, H. & Yushkevich, P. A. Groupwise segmentation with multi-atlas joint label fusion. *Med. Image Comput. Comput. Assist. Interv.* **16**, 711–718 (2013). 24505730 10.1007/978-3-642-40811-3_89 PMC3918678

[45] Isensee, F., Jaeger, P. F., Kohl, S. A. A., Petersen, J. & Maier-Hein, K. H. nnU-Net: a self-configuring method for deep learning-based biomedical image segmentation. *Nat. Methods* **18**, 203–211 (2021). 33288961 10.1038/s41592-020-01008-z

[46] Yushkevich, P. A. *et al*. User-guided 3D active contour segmentation of anatomical structures: significantly improved efficiency and reliability. *Neuroimage* **31**, 1116–1128 (2006). 16545965 10.1016/j.neuroimage.2006.01.015

[47] Wedig, M. M., Rauch, S. L., Albert, M. S. & Wright, C. I. Differential amygdala habituation to neutral faces in young and elderly adults. *Neurosci. Lett.* **385**, 114–119 (2005). 15961229 10.1016/j.neulet.2005.05.039

[48] Filipek, P. A., Richelme, C., Kennedy, D. N. & Caviness, V. S. Jr. The young adult human brain: an MRI-based morphometric analysis. *Cereb. Cortex* **4**, 344–360 (1994). 7950308 10.1093/cercor/4.4.344

[49] Rushmore, R. J. *et al*. Anatomically curated segmentation of human subcortical structures in high resolution magnetic resonance imaging: An open science approach. *Front. Neuroanat.* **16**, 894606 (2022). 36249866 10.3389/fnana.2022.894606 PMC9562126

[50] Feczko, E. *et al*. BOBsRepository. *OSF*10.17605/OSF.IO/WDR78 (2024).

[51] Feczko, E. *et al*. BOBsRepo. *OpenNeuro* https://openneuro.org/datasets/ds005450/ (2025).

[52] Standring, S. *Gray’s Anatomy: The Anatomical Basis of Clinical Practice*. (Elsevier, 2020).

[53] Morey, R. A. *et al*. A comparison of automated segmentation and manual tracing for quantifying hippocampal and amygdala volumes. *Neuroimage* **45**, 855–866 (2009). 19162198 10.1016/j.neuroimage.2008.12.033 PMC2714773

[54] Gorgolewski, K. J. *et al*. BIDS apps: Improving ease of use, accessibility, and reproducibility of neuroimaging data analysis methods. *PLoS Comput Biol* **13**, e1005209 (2017). 28278228 10.1371/journal.pcbi.1005209 PMC5363996

[55] Poldrack, R. A. *et al*. The past, present, and future of the brain imaging data structure (BIDS). *Imaging Neurosci (Camb)* **2**, 1–19 (2024). 39308505 10.1162/imag_a_00103 PMC11415029

[56] Creators Sturgeon, Darrick1 Snider, Kathy1 Moore, Lucille A. 2 Perrone, Anders J. 2 Earl, Eric3 Madison, Thomas J. 4 Conan, Greg4 Klein, Rachel Miranda-Dominguez, Oscar4 Feczko, Eric5 Graham, Alice1 Fair, Damien4 Show affiliations 1. Oregon Health and Science University 2. Masonic Institute for the Developing Brain 3. National Institute of Mental Health 4. Masonic Institute for the Developing Brain, University of Minnesota 5. (1) Masonic Institute for the Developing Brain, University of Minnesota & (2) Institute of Child Development, University of Minnesota. *DCAN-Labs Infant-Abcd-Bids-Pipeline*. 10.5281/zenodo.7683282.

[57] Edwards, A. D. *et al*. The Developing Human Connectome Project Neonatal Data Release. *Front. Neurosci.* **16**, 886772 (2022). 35677357 10.3389/fnins.2022.886772 PMC9169090

[58] de Macedo Rodrigues, K. *et al*. A FreeSurfer-compliant consistent manual segmentation of infant brains spanning the 0–2 year age range. *Front. Hum. Neurosci*. **9** (2015). 10.3389/fnhum.2015.00021 PMC4332305 25741260

